# Supplementary material for: Effects on Cell Membrane Integrity of Pichia anomala by the Accumulating Excessive Reactive Oxygen Species under Ethanol Stress
Source: Foods. 2022 Nov 21;11(22):3744. doi: 10.3390/foods11223744 (PMC9689904; doi:10.3390/foods11223744)
Supplement: Supplementary file 1 [file foods-11-03744-s001.zip › foods-2004010-supplementary.pdf]

Article

Effects on Cell Membrane Integrity of *Pichia anomala* by the Accumulating Excessive Reactive Oxygen Species under Ethanol Stress

Table S1 The result of produce flavor compounds of *P. anomala* NCU003 under the ethanol stress

	Flavor compounds	Result RI	Reference RI	References
1	Ethyl acetate	633	62	Fan et al. 2009
2	Ethyl propionate	719	714	Pino et al. 2005
3	Propyl acetate	720	717	Liu et al. 2007
4	Isobutyl acetate	746	749	Raffo et al. 2009
5	Isopentyl acetate	891	876	Pino et al. 2005
6	Isoamyl formate	893	880	Pino et al. 2005
7	n-Pentyl propionate	972	972	Javidnia et al. 2004
8	Furfuryl acetate	997	998	Kim et al. 2006
9	Ethyl hexanoate	1000	996	Pino et al. 2005
10	Etheyl octanoat	1197	1196	Pino et al. 2005
11	Phenylethyl acetate	1256	1256	Pino et al. 2005
12	Ethyl caprate/	1401	1397	Pino et al. 2005
13	Ethyl laurate	1648	1597	Mevy et al. 2006
14	Isoamyl alcohol	727	730	Jalali-Heravi et al. 2006
15	2-Methylbutan-1-ol	729	736	Pino et al. 2005
16	Phenylethyl alcohol	1117	1116	Alissandrakis et al. 2007
17	Benzaldehyde	962	966	Alissandrakis et al. 2007
18	Benzeneacetaldehyde	1040	1049	Alissandrakis et al. 2007
19	Nonanal	1103	1102	Asuming et al. 2005
20	Decanal	1205	1206	Mevy et al. 2006
21	Styrene	895	898	Pérez et al. 2007
22	Limonene	1025	1030	Harzallah-Skhiri et al. 2006

References

Fan G, Lu W, Yao X, Zhang Y, Wang K, Pan S (2009) Effect of fermentation on free and bound volatile compounds of orange juice. Flavour Fragr J 24(5): 219-229. https://doi.org/10.1002/ffj.1931.

Liu Y, Xu XL, Zhou GH (2007) Comparative study of volatile compounds in traditional Chinese Nanjing marinated duck by different extraction techniques. Int J Food Sci Technol 42(5): 543-550. https://doi.org/10.1111/j.1365-2621.2006.01264.x.

Jalali-Heravi M, Zekavat B, Sereshti H (2006) Characterization of essential oil components of Iranian geranium oil using gas chromatography-mass spectrometry combined with chemometric resolution techniques J Chromatogr A 1114(1): 154-163. https://doi.org/10.1016/j.chroma.2006.02.034.

Pino JA, Mesa J, Muñoz Y, Martí MP, Marbot R (2005) Volatile components from mango (*Mangifera indica* L.) cultivars. J Agric Food Chem 53(6): 2213-2223. https://doi.org/10.1021/jf0402633.

Raffo A, Kelderer M, Paoletti F, Zanella A (2009) Impact of innovative controlled atmosphere storage technologies and postharvest treatment on volatile compound production in Cv. *Pinova* apples. J Agric Food Chem 57(3): 915-923. https://doi.org/10.1021/jf802054y.

Pérez RA, Navarro T, de Lorenzo C (2007) HS-SPME analysis of the volatile compounds from spices as a source of flavour in 'Campo Real' table olive preparations. Flavour Fragr J 22(4): 265-273. https://doi.org/10.1002/ffj.1791.

Alissandrakis E, Tarantilis PA, Harizanis PC, Polissiou M (2007) Comparison of the volatile composition in thyme honeys from several origins in Greece. J Agric Food Chem 55(20): 8152-8157. https://doi.org/10.1021/jf071442y.

Javidnia K, Miri R, Sadeghpour H (2004) Composition of the volatile oil of *Achillea wilhelmsii* C. Koch from Iran. DARU, 12(2): 63-66. https://www.researchgate.net/publication/267818052.

Kim MR, Abd El-Aty AM, Kim IS, Shim JH (2006) Determination of volatile flavor components in danggui cultivars by solvent free injection and hydrodistillation followed by gas chromatographic-mass spectrometric analysis. J Chromatogr A 1116(1-2): 259-264. https://doi.org/10.1016/j.chroma.2006.03.060.

Harzallah-Skhiri F, Ben Jannet H, Hammami S, Mighri Z (2006) Variation of volatile compounds in two Prosopis farcta (Banks et Sol.) Eig. (Fabales, Fabaceae = Leguminosae) populations. Flavour Fragr J 21(3): 484-487. https://doi.org/10.1002/ffj.1652.

Asuming WA, Beauchamp PS, Descalzo JT, Dev BC, Dev V, Frost S, Ma CW (2005) Essential oil composition of four Lomatium Raf. species and their chemotaxonomy. Biochem Syst Ecol 33(1): 17-26. https://doi.org/10.1016/j.bse.2004.06.005.

Mevy JP, Bousquet-Melou A, Greff S, Millogo J, Fernandez C (2006) Chemical composition of the volatile oil of Laggera aurita Schulz from Burkina-Faso. Biochem Syst Ecol 34(11): 815-818. https://doi.org/10.1016/j.bse.2006.06.005.
